# Communication About OFF Periods in Parkinson's Disease: A Survey of Physicians, Patients, and Carepartners

**DOI:** 10.3389/fneur.2019.00892

**Published:** 2019-08-19

**Authors:** Tara Rastgardani, Melissa J. Armstrong, Anna R. Gagliardi, Arthur Grabovsky, Connie Marras

**Affiliations:** ^1^The Morton and Gloria Shulman Movement Disorders Centre and the Edmond J. Safra Program in Parkinson's Research, Toronto Western Hospital, University of Toronto, Toronto, ON, Canada; ^2^Department of Neurology, University of Florida College of Medicine, Gainesville, FL, United States; ^3^Toronto General Hospital Research Institute, University Health Network, University of Toronto, Toronto, ON, Canada; ^4^Tensor Consulting Services, Toronto, ON, Canada

**Keywords:** Parkinson's disease, fluctuations, “off” periods, communication, clinical care

## Abstract

**Background:** OFF periods impair quality of life in Parkinson's disease and are often amenable to treatment. Optimal treatment decisions rely on effective communication between physicians, patients and carepartners regarding this highly variable and complex phenomenon. Little is published in the literature about communication about OFF periods.

**Methods:** Informed by interviews with physicians, patients and carepartners we designed questionnaires for each group. We surveyed these parties using an online platform to investigate the frequency, content and ease of communication about OFF periods and barriers and facilitators of communication with physicians.

**Results:** Fifty movement disorder neurologists, 50 general neurologists, 442 patients and 97 carepartners participated. A free-flowing dialogue is the mainstay of communication according to all parties. Motor aspects of OFF periods are discussed more frequently than non-motor aspects (90 vs. <50% according to both general neurologists and movement disorder neurologists). The most common physician-reported barriers to communication are patient cognitive impairment, patient difficulty recognizing OFF periods and poor patient understanding of OFF periods' relationship to medication timing. The barriers most commonly cited as major by patients were that they perceived OFF periods to be part of the disease (i.e., not a clinical aspect that could be improved by a physician), variability of symptoms, and difficulty in describing symptoms. The most commonly described facilitator (by physicians) was the input of a caregiver. Positively viewed but less commonly used facilitators included pre-visit questionnaires or diaries, digital apps and wearable devices to monitor fluctuations. The majority of patients and carepartners identified a free-flowing dialogue with their physicians and having an agenda as helpful facilitators of communication about OFF periods which they already use. The majority of both groups felt that keeping a diary and pre-visit questionnaires were potentially helpful facilitators that were not currently in use.

**Conclusions:** Perceived barriers and facilitators to communication about OFF periods are different between health care providers and receivers of health care. Modifiable barriers and facilitators that could be implemented were identified by both groups. Future research should develop and test strategies based on this input to optimize communication and thus clinical care for this common and debilitating problem.

## Introduction

Parkinson's disease (PD) is a chronic progressive neurodegenerative condition which causes a wide array of motor, cognitive and emotional symptoms. OFF periods are a temporary re-emergence of symptoms of PD that are often controlled or partially controlled by medication. OFF periods are associated with poorer health-related quality of life in PD (1). The combination and severity of these symptoms are unique for each person with Parkinson's disease (PwP) and include a broad spectrum of motor and non-motor symptoms (2, 3). They are also heterogeneous in their temporal pattern and this can vary not only between PwP but from day to day (4). They are often amenable to treatment, but the complexity of OFF periods necessitates detailed and clear communication to understand the patient experience, inform treatment decisions and counsel patients.

Good communication between patients and health care providers is associated with better patient outcomes as well as patient satisfaction (5). The nature of communication between the physician and the patient is important to patients, who value candid and empathic communication and adequate time to communicate (6). A common source of patient dissatisfaction with physician services is failure to listen and understand the patient's feelings (7). A substantial proportion of patients wish to take the lead in decision-making (8), which necessitates explanations by the physician about the implications of the treatment options in the context of the patient's concerns. Carepartners often play an important role in supporting patients and often have a role in communications regarding the patient's condition at clinical visits, particularly in progressive neurodegenerative conditions like PD (9, 10).

A scoping review (1) found that there is a paucity of published information regarding communication about OFF periods between PwP or carepartners and treating physicians. One study was identified that assessed communication about OFF periods: A survey of PwP and their carepartners found that less than half of the PwP indicated that they discuss troublesome wearing *off* symptoms at every appointment (11). Only a quarter of carepartners indicated that they have ever discussed the impact of the PwP's symptoms on their own life and a similar proportion indicated that that they are not currently asked about the impact of troublesome symptoms on their own lives, but would like to be. Prior to the current mixed-methods study, no identified investigated barriers of communication about OFF periods. After investigating communication about OFF periods through preparatory interviews with PwP, carepartners, and physicians (12), we undertook a survey of PwP, carepartners, and physicians to understand the nature of communication about OFF periods and barriers and facilitators to communication from all three perspectives in a larger cohort. This knowledge is needed to understand the types of interventions that may be needed to support discussion about OFF periods.

## Materials and Methods

We administered an online cross-sectional survey to understand the opinions and experiences of American PwP, carepartners and physicians. We report our methods and results adhering to the STROBE checklist (http://www.equator-network.org/).

### Questionnaire Development

We developed questionnaires (see Supplementary Material) concerning the experience, understanding and communication about OFF periods based on data collected in qualitative interviews with representatives of each of the three groups (PwP, carepartners and physicians). The interviews (results reported separately) (12), were performed to ensure that questionnaire content covered the main issues, concerns and opinions facing these three groups. The questionnaire for physicians addressed their communication practices about OFF periods with PwP and their opinions on barriers and facilitators of this communication. The questionnaire for PwP and carepartners addressed the symptoms and impact of OFF periods, as well as their opinions on barriers and facilitators of communication about OFF periods with physicians. The potential barriers and facilitators presented to PwP and carepartners were the same, but physicians were presented with different potential barriers and facilitators, based on the insights we obtained from the qualitative interviews preceding this survey-based study. Questionnaires were not tested for psychometric properties because the aim of the survey was descriptive and not analytic. The questionnaires were pilot tested by several PwP and carepartners from the practice of one of the authors (CM) as well as several neurologists to ensure comprehension and sound logic. All feedback was reviewed by the authors and incorporated into the questionnaire.

### Sampling and Recruitment

PwP and carepartners were recruited through Fox Insight (https://foxinsight.michaeljfox.org/), an online data collection platform developed and maintained by the Michael J Fox Foundation. This platform allows PwP and interested individuals without Parkinson's disease to contribute longitudinal data on their medical conditions, quality of life and lifestyle. Platform participants are invited by email to participate in ancillary projects, and eligible Fox Insight participants were sent an invitation to complete our questionnaires. The questionnaires were open in Fox Insight from February 7th, 2018 to March 30th, 2018. Up to 2 repeated invitations were sent at 4 and 7 weeks after the first invitation if individuals did not open the email. For the current study, Fox Insight participants were eligible to participate if they self-reported a diagnosis of Parkinson's disease, were on treatment for PD (not necessarily levodopa), and reported the occurrence of OFF periods. No further diagnostic confirmation was possible. Carepartners were eligible to participate if they were the primary caregiver for someone who currently experienced OFF periods. Patients and carepartners were not recruited as dyads. The following explanation of OFF periods was provided: “*When a person with Parkinson's disease benefits from medication, over time they can begin to experience episodes where the medications don't work or don't work as well. In those episodes those symptoms that are typically improved by the medication temporarily worsen. These episodes are called* ***OFF*** ***periods***.”

Physicians were recruited through Sermo, a physician social network that also invites physicians to participate in clinical research (http://www.sermo.com). Sermo sent invitations by email to its membership, inviting neurologists seeing at least 10 patients with Parkinson's disease per month to participate. Invited physicians received a single invitation, over a recruitment period of February 22nd to March 9th 2018.

### Data Collection

Surveys for PwP and carepartners were administered online through the Fox Insight platform using a custom software. The physician survey was administered online using Confirmit software. For all three groups of participants answers were required to proceed to the next question. Respondents could complete their questionnaire in multiple sittings. Data entry into a database was accomplished directly via the software program.

### Bioethics

After an introductory statement explaining the nature and length of the questionnaire, proceeding with the questionnaire was taken as implied consent to participate. Written consent was not obtained. The protocol was approved by the Research Ethics Board of the University Health Network (Toronto, Canada).

### Statistical Analysis

Frequencies were summarized as medians and interquartile ranges. Associations between potential barriers or facilitators of communication and age and duration of disease were tested using logistic regression models.

## Results

### Descriptive Characteristics of Samples

#### PwP and Carepartners

PwP/carepartner response rate was 13% (2,262/17,085). Due to an initial programming error precluding linkage to the main Fox Insight dataset, data from the first 1,727 respondents were invalid. Thus, responses from 442 PwP and 97 carepartners were included. There were no significant demographic or disease duration differences between those included and excluded (mean age 65 years, 51% female for both groups). The 539 included (PwP + carepartners) were of similar age (mean 66 years) compared with the 15,823 non-respondents (mean age 63 years). 93% of both respondents and non-respondents had post-secondary education. The ratio of men to women was similar for respondents (51% women, 46% men 3% not reported) and non-respondents (42% women, 37% men, 21% not reported) and mean disease duration was similar as well (5 years for both respondents and non-respondents). The demographic characteristics and the characteristics of the OFF periods of those included are shown in Table 1.

**Table 1:** Characteristics of participating people with Parkinson's disease and carepartners.

	**PwP**	**Care partners**
Number	442	97
Median age (IQR)	66 (60, 71)	64 (60, 69)
% male	51	24
Median disease duration, years (IQR)	5 (2, 8)	N/A
Disease duration of the person with PD, n		
1–5 years	41	
6–10 years	28	
11–15 years	14	
16–20 years	8	
>20 years	6	
Type of physician seen		
Movement disorder specialist	271	56
General neurologist	159	34
Primary care physician	8	4
Other	2	3
Unknown to participant	2	0
Median frequency of OFF periods (number per day)	2	2
Median duration of each OFF periods	30–45 min	30–45 min

*PwP, People with Parkinson's disease*.

#### Neurologists

Neurologist response rate was 16% (223/1393). Respondents were of similar sex distribution to non-respondents (74 and 76% male, respectively), but respondents were younger (mean age 45 years) than non-respondents (mean age 57 years). Among interested respondents, 123 did not qualify and were excluded. Fifty movement disorders physicians and 50 general neurologists completed the physician survey. For both physician types the median number of years in practice was more than 10.

### Communication With Health Care Professionals About OFF Periods

PwP and carepartners were asked to recall what aspects of OFF periods were discussed at the office visit. Discussion of motor aspects was reported by a higher proportion of both groups (PwP 57%, carepartners 89%) than non-motor aspects (emotional symptoms: PwP 30%, carepartners 56%, bodily functions, e.g., urinary symptoms, sweating, hot flashes: PwP 22%, carepartners 41%). A higher proportion of carepartners than PwP reported discussion of all aspects. Fifty nine percent of carepartners and 79% of PwP felt that the doctor understood the burden of OFF periods on their lives. Motor aspects of OFF periods were also the aspect most frequently reported as discussed by physicians (90% for both general and movement disorder neurologists), followed by the impact of OFF periods (on PwP: 80% for general neurologists, 86% for movement disorder neurologists) and on carepartners: 56% of general neurologists and 64% of movement disorder neurologists), followed by non-motor aspects (<50% for both groups).

The clinical interview was used by all neurologists to assess OFF periods. Including carepartners in the clinical interview was a frequent complementary method (general neurologists 86% and movement disorder 88%). The frequency of use of other methods such as questionnaires, diaries, wearable devices or direct observation are shown in Table 2.

**Table 2:** Percentage of general and movement disorder neurologists using each strategy for facilitating communication of OFF symptoms.

**Strategy**	**General neurologists (*****n*** **=** **50)**		**Movement disorder neurologists (*****n*** **=** **50)**	
	Sometimes^*^ (%)	Always or almost always^*^ (%)	Sometimes^*^ (%)	Always or almost always^*^ (%)
Speaking to patient during clinical interview	6%	94%	2%	98%
Speaking to care partner during clinical interview	12%	86%	10%	88%
Using a questionnaire that the patient and/or care partner completes prior to clinical interview	28%	36%	32%	40%
Using a motor diary that patient and/or care partner completes at home or prior to clinic visit	28%	38%	18%	48%
Levodopa challenge observing a patient through a dose-response cycle of levodopa administration	34%	32%	28%	36%
Using wearable technology (e.g., a device that collects data on patterns on movement of the patient at home, which may be analyzed for periods of tremor, lack of movement, or dyskinesia)	16%	6%	6%	16%

* *Assessed on a Likert scale where 1, Never; 5, Always. 2 or 3 is reported as Sometimes, 4 as Almost always*.

### Education About OFF Periods

The most commonly reported sources of education about OFF periods by PwP were online resources (57%), books (31%), or physicians (56%). This was similar for carepartners (see Supplementary Table 1) with the exception of a higher proportion learning about OFF periods through support groups (23 vs. 11% for patients). Sixty three percent of patients and 69% of carepartners reported that they were satisfied with the education regarding OFF periods that they had received from their physicians.

### Barriers to Communication About OFF Periods

**Figure 1:**
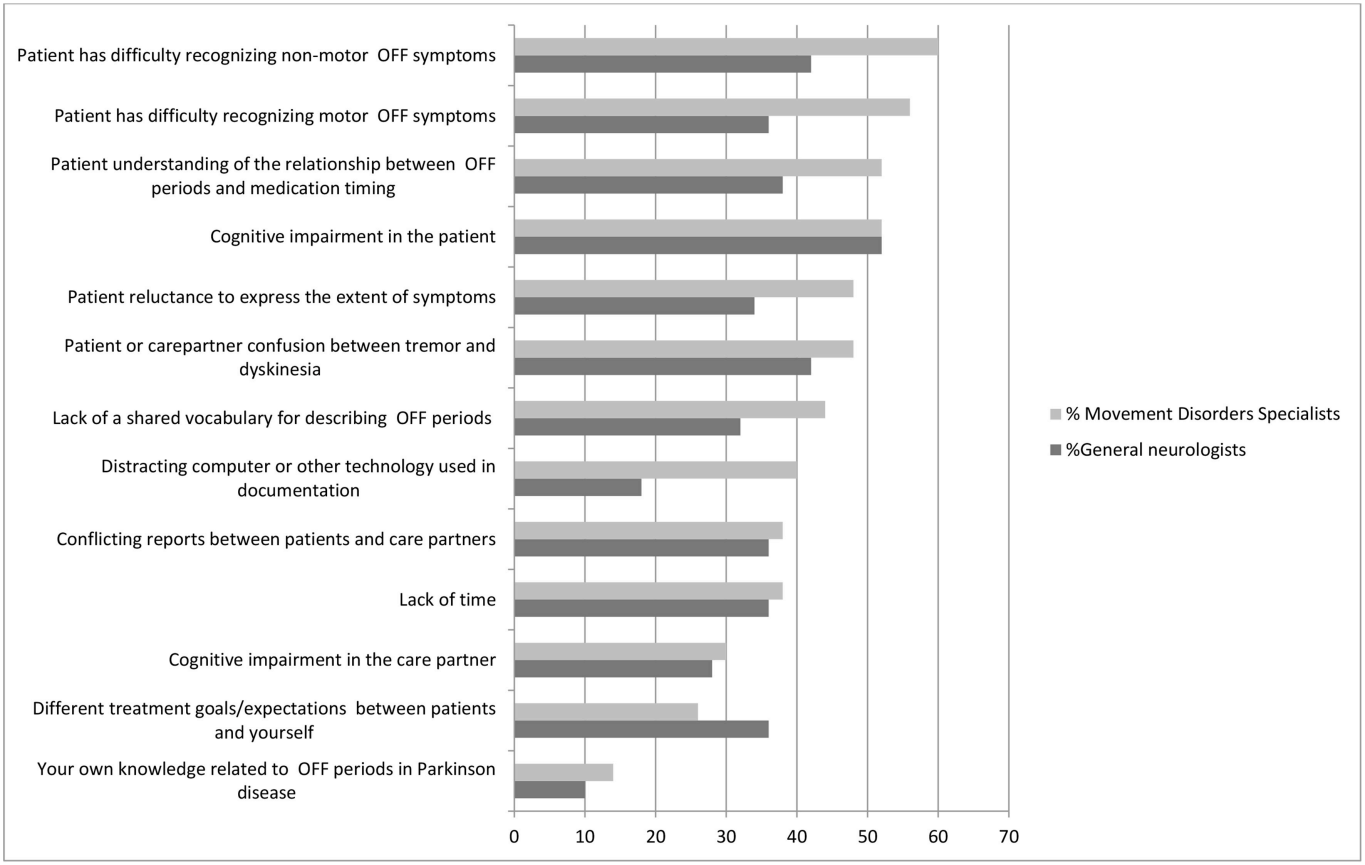
Physician-reported barriers to communication about OFF periods.

Physicians noted a number of major barriers to communication regarding OFF periods in the office (Figure 1). According to movement disorder physicians, difficulty on the part of the patient recognizing motor and non-motor symptoms of OFF, poor understanding of the relationship between OFF periods and medication timing and cognitive impairment in the patient were the most commonly cited major barriers. According to general neurologists, cognitive impairment in the patients, confusion between dyskinesias and tremor and difficulty recognizing motor symptoms of OFF were most commonly cited as major barriers.

**Figure 2:**
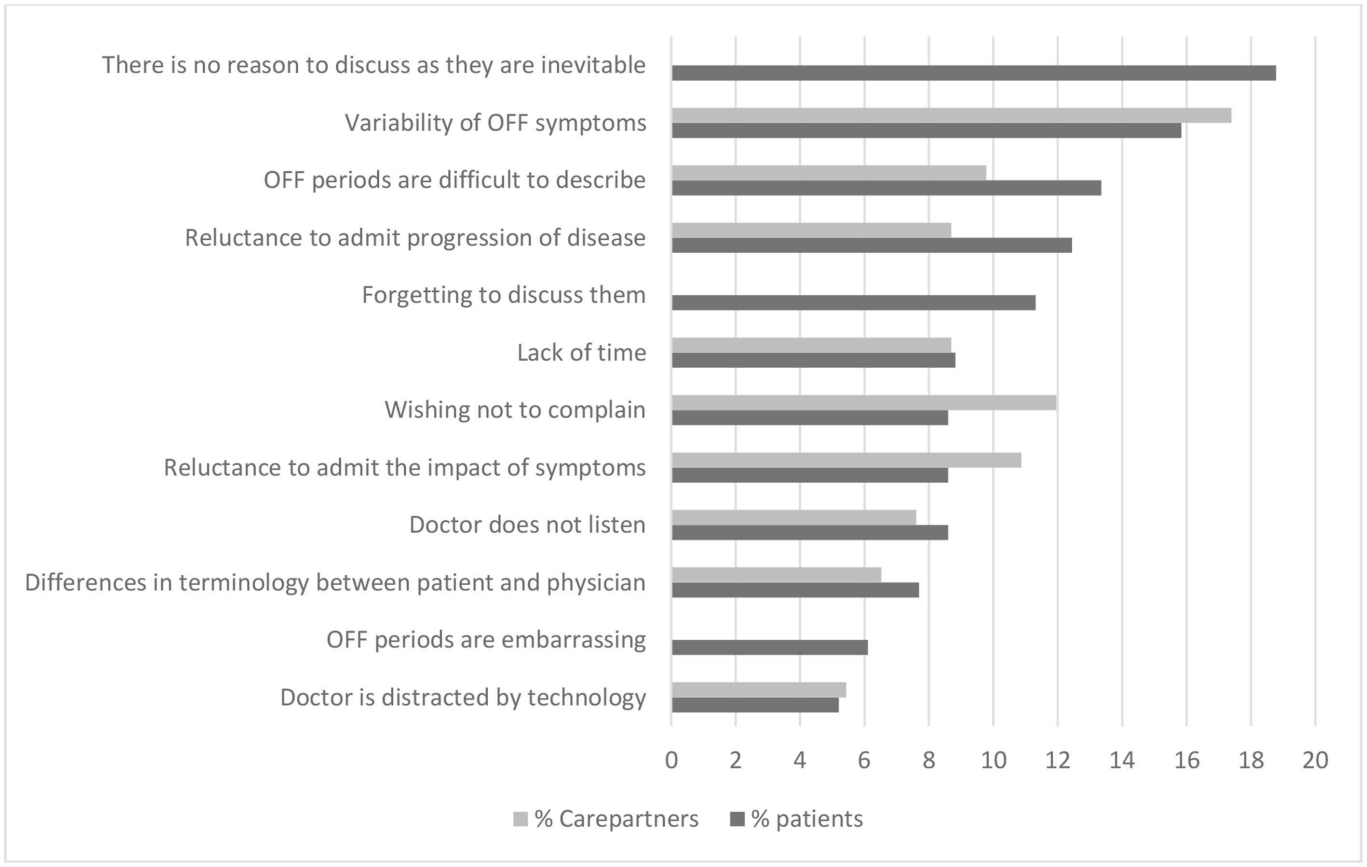
Barriers to communication about OFF periods reported by PwP and carepartners.

Patients and carepartners also noted barriers to communication about OFF periods (Figure 2). The barriers most commonly cited as major by patients were that they were simply felt to be part of the disease (i.e., not a clinical aspect that could be improved by a physician), variability of symptoms, and difficulty in describing symptoms. Carepartners most commonly identified variability of OFF symptoms, patient reluctance to “complain,” and patient reluctance to admit the impact of the OFF periods on their lives as major barriers to communication about OFF periods. Patients and carepartners differed most according to the impression that OFF periods are inevitable, cited as a major barrier by 19% of patients and none of the carepartners. We did not find any significant association between age or disease duration and the cited barriers.

### Facilitators of Discussing OFF Periods

Physician ratings of facilitators to the discussion of OFF periods are shown in Table 3. The facilitator most commonly identified by both general neurologists and movement disorders specialists was the presence of a care partner at the clinical visit (90 and 92%). A high percentage of general neurologists and movement disorders specialists also already used this strategy for communication (82 and 84%, respectively). Free-flowing dialogue was the next most commonly reported facilitator in both groups (88 and 84%). Again, a high percentage of both physician groups reported already using this method for communication (76 and 72%). Incorporating a teach-back technique in communication was identified as a facilitator by a high percentage (62 and 78%), but was less commonly implemented as a method of communication (40 and 56%). A multidisciplinary approach incorporating allied health personnel such as nurse educators was seen as a facilitator by both groups (64 and 66%), but less used by general neurologists than movement disorders specialists (28vs. 50%).

**Table 3:** Facilitators of communication about OFF periods cited by physicians.

**Facilitator**	**General neurologist**		**Movement disorder neurologist**	
	**Theoretically helpful or very helpful *n* (%)**	**Using this strategy *n* (%)**	**Theoretically helpful or very helpful *n* (%)**	**Using this strategy *n* (%)**
Presence of a care partner at the clinical visit	45 (90%)	41 (82%)	46 (92%)	42 (84%)
Free-flowing dialogue	44 (88%)	38 (76%)	42 (84%)	36 (72%)
Access to allied health personnel (e.g., nurse educator)	32 (64%)	14 (28%)	33 (66%)	25 (50%)
Information pamphlet	29 (58%)	17 (34%)	31 (62%)	19 (38%)
Diagram-based explanation of OFF periods to facilitate discussion	26 (52%)	12 (24%)	26 (52%)	15 (30%)
On-line video	26 (52%)	4 (8%)	27 (54%)	14 (28%)
Pre-consultation questionnaire	21 (42%)	14 (28%)	28 (56%)	22 (44%)
Paper-based motor diaries to be completed at home	24 (48%)	16 (32%)	25 (50%)	21 (42%)
Digital app on smart phone to record OFF time	27 (54%)	7 (14%)	25 (50%)	11 (22%)
Wearable technology to automatically detect OFF time	30 (60%)	5 (10%)	31 (62%)	10 (20%)
Levodopa challenge^*^	33 (66%)	25 (50%)	32 (64%)	26 (52%)
Home videos provided by patients and/or care partners	29 (58%)	14 (28%)	33 (66%)	20 (40%)
Teach-back method^**^	31 (62%)	20 (40%)	39 (78%)	28 (56%)
Small group classes	20 (40%)	7 (14%)	25 (50%)	8 (16%)
Repeated educational points over multiple visits	43 (86%)	38 (76%)	34 (68%)	29 (58%)
Other	0 (0%)	0 (0%)	1 (2%)	0 (0%)

* *Observing a patient through a dose-response cycle of levodopa administration*.

** *Having patients and/or care partners repeat back information to ensure comprehension*.

Facilitators of communication as reported by patients and carepartners are shown in Table 4. The majority of patients and carepartners identified a free-flowing dialogue with their physicians and having an agenda as helpful facilitators of communication about OFF periods which they already use. Additionally, 48 (9%) of PwP additionally offered education about OFF periods as a helpful facilitator of communication, and they had usually obtained this from web-based sources. The majority of patients felt that keeping a diary, taking a home video and pre-visit questionnaires were potentially helpful facilitators but were not currently in use. The majority of carepartners identified keeping a diary, pre-visit questionnaires and wearable devices as potentially helpful strategies that they do not currently use. Age of the patient was not significantly related to the odds of reporting any of the potential facilitators as being helpful. Longer disease duration was associated with reduced odds of reporting a longer clinic visit to be helpful (OR per 5 year increment in disease duration = 0.7, 95% CI 0.6–0.8), and reduced odds of reporting questionnaires to be helpful (OR 0.7 per 5-year increment, 95% CI 0.6–0.8).

**Table 4:** Facilitators of communication as reported by patients and carepartners.

	**Patients (*****n*** **=** **442)**		**Carepartners (*****n*** **=** **97)**	
	**I have used this and it is helpful *n* (%)**	**I think this would be helpful if i tried it *n* (%)**	**I have used this and it is helpful *n* (%)**	**I think this would be helpful if i tried it *n* (%)**
Free-flowing dialogue	272 (61)	125 (28)	64 (70)	23 (25)
Having an agenda	259 (58)	141 (31)	67 (73)	22 (24)
Longer visits	169 (28)	149 (33)	44 (48)	**31** (34)
Having carepartner describe OFF periods	157 (36)	76 (17)	**n/a**	**n/a**
Keeping a diary	108 (24)	268 (61)	24 (26)	56 (61)
Pre-visit questionnaires	99 (22)	267 (60)	23 (25)	51 (55)
Taking a home video	49 (11)	280 (63)	12 (13)	41 (44)
Wearable devices	0 (0)	187 (42)	12 (13)	62 (67)
Other	66 (14)^*^	147 (33)	15 (16)^**^	17 (18)

* *Education about OFF periods acquired online (n = 36), education about OFF periods (method not specified) (n = 9), educating family members about OFF periods (n = 3), patient portal for communication with physician (n = 3), support groups (n = 3), education from handbooks (n = 1), education from other health professionals (n = 1), unspecified (n = 10)*.

** *Education about OFF periods acquired online (n = 3), educational written materials about OFF (n = 2), email or text messages with physician (n = 3), talking with the PwP (n = 2), group discussion (n = 2), discussion with other family members (n = 1), making notes in smart phone (n = 1), patient portal for communication with physician (n = 1). n/a, not applicable*.

## Discussion and Conclusions

Our study has revealed that communication about OFF periods in the physician's office is based on a free-flowing narrative, with a small minority using questionnaires or other tools to gather information. Physicians, patients and carepartners all perceive that motor aspects of OFF periods are more frequently discussed than non-motor aspects. The awareness of non-motor aspects of OFF periods has emerged more recently. A questionnaire assessing non-motor fluctuations has been developed but has yet to be fully psychometrically evaluated (13). The inquiry of multiple facets of OFF periods and understanding their relative impact on the PwP by way of a questionnaire is complex and this task may lend itself more readily to a free-flowing dialogue. The content of our questionnaire did not include inquiry of the reasons for the low uptake of such instruments by physicians. Understanding the reasons for this low uptake would be a useful avenue to pursue in order to understand whether or not questionnaires hold promise for improving communications surrounding OFF periods, particularly in the non-motor domain.

Our inquiry about barriers and facilitators of communication about OFF periods revealed a number of potential avenues to improve the clinical interaction. A poor understanding of the relationship between OFF periods and medication timing, confusion between tremor and dyskinesia, and poor recognition of motor and non-motor aspects of OFF periods were identified barriers that could be addressed by educational efforts. The lack of a shared vocabulary to describe and discuss OFF periods was also cited as a barrier by all three groups and could be addressed by an effort on the part of the physician to establish a common vocabulary in the office. This information corresponds well with the suggested facilitators, that are an educational pamphlet, online video, small group classes or a diagram portraying fluctuations over the course of several medication cycles. While there are educational resources about understanding and managing symptoms of Parkinson's disease available from charitable foundations, there is relatively little information devoted to educating on the nature and management of OFF periods. Resources for facilitating the understanding of OFF periods include a diagram portraying fluctuations and the relationship to medication such as can be found accompanying the Wearing Off Questionnaire (14). Paper-based symptom tracking tools exist, such as that found in the Parkinson's Foundation “Caring and Coping, a Caregiver's Guide to Parkinson's disease” (http://www.parkinson.org/sites/default/files/attachments/Caring_and_Coping.pdf), or the instrument developed as part of the SCOPA project (15), however their ability to convey the symptom and temporal complexity of OFF symptoms is limited.

The presence of a carepartner at the office visit was valued as a facilitator by the vast majority of physicians, and was also one of the more commonly cited facilitators by patients. It is interesting that a higher proportion of carepartners reported recalling discussion of all aspects of OFF periods than patients, suggesting incomplete recall on the part of more patients and supporting the value of carepartner involvement. Our qualitative interviews also support the perceived value of carepartner input by all parties and the desire for carepartners to be part of the discussion (16). It was clear from those interviews, however, that they are often not included in the conversation. A lack of engagement of carepartners at the clinical interviews has been reported in previous research as well (10, 17). Physicians can encourage carepartners to attend visits and be mindful of including them when appropriate.

Technological solutions such as digital applications for recording OFF periods or wearable technology to record fluctuations were identified as likely useful by a majority of physicians but used by only a few, indicating an opportunity for improving clinical care. Wearable technology is in development or available to record motor features of the disease particularly tremor, gait and activity levels. (e.g., http://oneringforpd.com/) (18, 19). Less well developed is the measurement of non-motor features or general well-being, although these aspects are under investigation (20). Free smart phone apps have been developed to measure tremor and to monitor other aspects of the disease through self-report or testing maneuvers (e.g., PD Me). These strategies can continuously and objectively monitor motor parameters, although robust validation of their measurement properties is awaited and the ability to capture many of the non-motor symptoms of OFF periods is limited (21).

The most commonly cited barrier to communication about OFF periods by patients was the perception that they are “part of the disease.” This reflects a concerning fatalistic attitude toward fluctuations, the perception that they are not amenable to treatment and therefore not worth discussing with the physician. This perception is congruent with the reporting by physicians that patients do not understand the relationship between medication timing and fluctuations, and may respond to education on this point. Also concerning is the reporting by patients that they are reluctant to discuss OFF periods because they represent progression of the disease. This may represent conscious avoidance, suppression or disavowal. Such avoidance or denial of illness or its severity has been identified as a barrier to health-care seeking in other health conditions (22–24). Physicians need to be alert to this tendency and patients may benefit from being directed toward sources of support or counseling on coping strategies (24).

The variability of OFF periods and difficulty describing OFF periods are cited barriers that may benefit from a questionnaire that prompts the patient about possible symptoms and aspects of OFF periods. Screening questionnaires and severity scales measuring OFF periods were reviewed in a Movement Disorder Society commissioned review (25). The WOQ-19 (14) and WOQ-9 (26) were rated as recommended screening instruments, and motor fluctuation diaries and the CAPSIT PD diary were rated as recommended severity scales based on their features and clinimetric testing. The Movement Disorders Society Unified Parkinson's Disease Rating Scale (MDS-UPDRS) (27) was deemed to have desirable features for a measurement tool of OFF periods but awaits clinimetric testing. The challenges capturing the complexities of OFF periods including variability of severity, duration and slow transitions between ON and OFF states were acknowledged as a limitation of these instruments. Such complexity may in the future be overcome by wearable devices that can monitor continuously and measure these aspects more precisely. The most extensively studied questionnaire for reporting OFF periods is the WOQ-19, which has been shown to identify more OFF periods than the clinician interview (28) and in its Spanish version has shown high positive and negative predictive value for identifying fluctuations when the clinician interview is considered to be the gold standard.

Despite the need to help patients to describe OFF periods and the opinions of physicians that strategies such as pre-visit questionnaires and diaries may be helpful facilitators, all parties seem to regard the free-flowing dialogue as the cornerstone of the clinical assessment of OFF periods. A combined approach (questionnaires or diaries complementing the dialogue) may be optimal, given that there is evidence that the clinical interview misses OFF periods detected by questionnaires (28). A not insignificant minority (5%) of patients regarded technology (e.g. the physician's computer) as a distractor from the interaction with their physician, and time is limited. Thus, it is important that whatever facilitator is used not present a significant additional time requirement that compromises the narrative history-taking. Completion before the visit and quick interpretation by the physician will thus be key attributes.

Strengths of our study include the large sample of patients and the recruitment of patients and carepartners through an online platform that does not restrict the sample to a specific geographic area or to those attending tertiary care centers. On the other hand, recruitment of patients and carepartners through an online platform is likely to select for highly motivated, highly educated individuals that may not be representative of the general population with Parkinson's disease. Indeed the median disease duration of approximately 6 years is also a relatively early group for a study of OFF periods and are likely experiencing more mild fluctuations than most. Individuals with more advanced disease and more severe fluctuations may have different attitudes and challenges related to communication about OFF periods. Another limitation of our study is that the diagnosis of PD relies on self-report by the participants, and thus it is possible that our sample includes some individuals without PD or with other conditions causing parkinsonism. The fact that we required individuals to be on treatment for PD and to experience fluctuations related to medication timing will likely reduce the chance of individuals without PD participating to a small number, but this cannot be verified by our methods. The response rate to the surveys was relatively low, but basic demographic characteristics of respondent and non-respondents were similar except for a tendency for participating physicians to be younger than non-participants. Nonetheless, generalizability of our results to the general population of PD and carepartners is uncertain. In addition, we did not recruit carepartners and patient dyads, therefore differences in the responses between these groups could reflect differences in the nature of the OFF periods that they have been experiencing or exposed to. However, as discussed above, the descriptions of frequency and duration provided by the two groups are similar. Finally, the description of OFF periods and the interactions between physicians and patients and carepartners may be affected by recall, as we did not require that the questionnaire be completed shortly after a visit to the physician.

In conclusion, our work has identified a number of barriers and facilitators of communication about OFF periods relevant to patients with relatively early PD and allows us to prioritize these based on the frequency with which they are reported by physicians, patients and carepartners. From these data potential avenues for improving communication can be identified, including educational videos or written material explaining OFF periods and the usual medical terminology relating to them, pre-visit questionnaires or diaries, and efforts by the physician to align vocabularies between the parties. Across all of this, involvement of the carepartner, even when the patient does not appear to have significant cognitive impairment, will promote a comprehensive understanding of the situation by the physician.

## Data Availability

The datasets generated for this study are available on request to the corresponding author.

## Ethics Statement

This study was carried out in accordance with the recommendations of the Research Ethics Board of the University Health Network, Toronto, Canada. Consent from all subjects was implied for all subjects by self-initiation of the survey after reading an introductory statement of the nature of the questionnaire and the time involved. The protocol was approved by the Research Ethics Board of the University Health Network.

## Author Contributions

CM, AGa, and MA: study conception and design. TR: data collection. AGa, CM, AGr, MA, and TR: data interpretation and analysis. CM: manuscript first draft. TR, MA, and AGa: manuscript review and approval.

### Conflict of Interest Statement

CM received speaker honoraria from Acorda Therapeutics. The remaining authors declare that the research was conducted in the absence of any commercial or financial relationships that could be construed as a potential conflict of interest.
