# Supplementary material for: Communication About OFF Periods in Parkinson's Disease: A Survey of Physicians, Patients, and Carepartners
Source: Front Neurol. 2019 Aug 19;10:892. doi: 10.3389/fneur.2019.00892 (PMC6709650; doi:10.3389/fneur.2019.00892)
Supplement: Supplementary file 1 [file Data_Sheet_1.docx]

Supplementary Table 1: Sources of education about OFF periods cited by people with Parkinson’s and carepartners

n/a=not applicable

| **Source** | **PwP**  **n (%)** | **Carepartners**  **n (%)** |
| --- | --- | --- |
| Physician | 250 (57) | 43 (44) |
| Carepartner | 12 (3) | n/a |
| Patient | n/a | 16 (16) |
| Internet | 251 (57) | 48 (49) |
| Book | 138 (31) | 36 (37) |
| Support group | 47 (11) | 22 (23) |
| Friend | 24 (5) | 2 (2) |
| Other | 40 (9) | 10 (10) |

PwP=people with Parkinson’s disease
