# Supplementary material for: Communication About OFF Periods in Parkinson's Disease: A Survey of Physicians, Patients, and Carepartners
Source: Front Neurol. 2019 Aug 19;10:892. doi: 10.3389/fneur.2019.00892 (PMC6709650; doi:10.3389/fneur.2019.00892)
Supplement: Supplementary file 2 [file Data_Sheet_2.docx]

**Impact and communication about OFF periods**

This survey seeks to understand how patients and care partners understand and communicate about OFF periods, and the impact that OFF periods have on patients and care partners. The survey should take about 25 minutes to complete.

**SCREENING QUESTION**

**Do you have Parkinson’s disease or are you the primary care partner for a person with Parkinson’s disease?**

1. **Yes I have Parkinson’s disease (direct to PD questionnaire)**
2. **Yes, I am the primary care partner for a person with Parkinson’s disease (direct to the carepartner questionnaire)**
3. **No (terminate)**

When a person with Parkinson’s disease benefits from medication, over time they can begin to experience episodes where the medications don’t work or don’t work as well. In those episodes those symptoms that are typically improved by the medication temporarily worsen. These episodes are called **OFF periods**.

1. Does the person you care for with Parkinson’s disease experience OFF periods, as just defined?
   1. Yes
   2. No **(TERMINATE)**
   3. I don’t know **(TERMINATE)**
2. Are you familiar with the term “OFF” or “OFF periods” related to Parkinson’s disease, as defined above?
   1. Yes
   2. No

**PERSONAL EXPERIENCE OF OFF PERIODS**

1. How long has the person you care for had Parkinson’s disease (in years)?
2. 1-5
3. 6-10
4. 11-15
5. 16-20
6. Greater than 20
7. I don’t know
8. How many years ago did the person you care for with Parkinson’s disease begin to experience OFF periods?

a)less than 1 year

b) 1 to 5 years

c) 6 to 10 years

d) Greater than 10 years

e) I don’t know

1. Over the last week, on average how many OFF episodes does the person you care for experience in a typical waking day?
   - Single Select (Radio)
     - No episodes, zero
     - 1 episode per day
     - 2 episodes per day
     - 3 episodes per day
     - 4 episodes per day
     - Greater than 4 episodes per day
     - I don’t know
2. Over the last week, on average what is the typical duration of each OFF episode?
   - Single Select (Radio)
     - Less than 15 minutes
     - Between 15 and 30 minutes
     - Between 30 minutes and 45 minutes
     - Between 45 minutes and 1 hour
     - Between 1 hour and 2 hours
     - Greater than 2 hours
     - I don’t know
3. What proportion of the OFF periods come at unpredictable (i.e. unexpected) times?
   1. 0
   2. Less than 25%
   3. 25-50%
   4. Greater than 50%
   5. I don’t know
4. If >0: If the timing of the OFF periods were more predictable, how much would that lessen their impact on your life?
   1. Very much
   2. Somewhat
   3. Neutral
   4. Not at all
5. Do you currently help the person you care for (who has Parkinson’s disease) keep track of his or her OFF periods using a paper or electronic record?
6. Yes
7. No

If yes, how do you keep track of OFF periods? (select all that apply)

1. Paper record
2. Electronic diary
3. He or she has a wearable device that I help manage
4. Other (please specify) _____________________________________
5. Check all of the symptoms of OFF periods experienced by the person you care for with Parkinson’s disease

| **Symptom** | **Yes/No/Unsure** |
| --- | --- |
| 1. Fatigue |  |
| 1. Sleepiness |  |
| 1. Tremor |  |
| 1. Stiffness |  |
| 1. Slowness of movement |  |
| 1. Change in gait/walking |  |
| 1. Increased falls |  |
| 1. Difficulty with hand coordination |  |
| Difficult swallowing |  |
| 1. i. Difficulty speaking |  |
| 1. Trouble breathing |  |
| 1. Nausea |  |
| 1. Pain |  |
| 1. Anxiety |  |
| 1. Irritability |  |
| 1. Agitation or restlessness |  |
| 1. Loss of motivation |  |
| 1. Sadness/depression |  |
| 1. Social withdrawal |  |
| 1. Hot flashes |  |
| 1. Sweating |  |
| 1. Loss of appetite |  |
| 1. Change in bladder function (e.g., urgency, incontinence) |  |
| 1. Difficulty thinking |  |
| 1. Other, please specify: ______ |  |

For each symptom indicated as “yes” need to follow with

A 5-point likert scale of impact:

1: No impact

5: Severe impact

**PERSONAL IMPACT OF OFF PERIODS**

**These questions are asking how the OFF times impact *your* life. Please answer these questions with respect to the impact on you, not the person with Parkinson’s disease.**

1. In general, how much impact do the OFF periods have on YOUR daily life?

5-point likert scale:

1=no impact

5=Severe impact

1. How much are each of the following aspects of YOUR life impacted by OFF periods ?Need a likert scale for each symptom ranging from

1=no impact

To 5=severe impact

|  |
| --- |
|  |
| Your Leisure/hobbies |
| Your Employment |
| Your Close Relationships |
| Your Friendships |
| Your Household tasks |
| Your Mood |
| Your Self-care |
| Your Physical Health |
| Your freedom to leave the home |
| Scheduled activities |

1. Does the person that you care for with Parkinson’s disease rely on you for timely administration of medication? (yes/no)
   1. If yes, Please rate the impact of that aspect on your life (likert scale from 1=no impact to 5=severe impact)
2. Using a scale from 1 to 7 (where 1=Strongly Disagree and 7=Strongly Agree) please rate your level of agreement with the following statements:
3. The OFF periods frustrate me
4. The OFF periods make me anxious
5. Watching OFF periods is scary
6. The OFF periods make me feel embarrassed

**EDUCATION ABOUT OFF PERIODS**

1. How did you learn about OFF periods? (select all that apply)
2. The doctor of the person I care for had told me about them before the person I care for began to experience them
3. The doctor of the person I care for explained that they were OFF periods after I brought up the symptoms
4. The person I care for explained it to me
5. I read about them on-line
   1. Which online source? ______
6. I read a book about Parkinson’s disease that explained what these symptoms were
   1. Which book? _____
7. The Parkinson’s disease support group told me what was happening
8. I learned about it from a friend
9. Other, please specify: _____
10. Do you feel that you have had adequate education about OFF periods? (e.g. teaching from your doctor, or educational material made available to you)
    1. Yes
    2. No
11. What would be your preferred format for learning about OFF periods
    1. Explanation from the doctor
    2. Written handout or pamphlet
    3. Explanation from the doctor + written handout
    4. On-line video tutorial
    5. On-line written material material
    6. Other (please specify) ___________________________________-
12. Do you think it is helpful to learn about OFF periods early in the course of Parkinson’s disease, before they are experienced?

(yes/No)

**COMMUNICATION REGARDING OFF PERIODS**

The following questions relate to communication about OFF periods with the doctor who takes care of most of the Parkinson’s disease-related issues.

**What type of doctor primarily cares for the person’s Parkinson’s disease?**

Movement Disorder specialist

General neurologist

Primary care physician

Geriatrician

Other

I don’t know

1. Do you attend appointments with the person who has Parkinson’s disease?

Yes/No/Sometimes

If Yes or Sometimes, proceed with highlighted questions:

1. How important do you feel it is for you as a caregiver to attend the appointments so that you can provide information to the doctor about the OFF periods? (e.g. the frequency, duration or impact of the OFF periods)

Likert scale: 1=Not important, to 5=very important

**IF YES:** What aspects of OFF periods are discussed at most visits:

| **Aspects of OFF periods** | **Discussed with the doctor (Yes/no)** |
| --- | --- |
| Frequency |  |
| Motor symptoms (e.g., tremor, stiffness, slowness of movement) |  |
| Emotional symptoms (e.g., anxiety) |  |
| Symptoms of bodily function (e.g., urinary symptoms, sweating, hot flashes) |  |
| Timing of OFF periods |  |
| Impact of OFF periods on the life of the person you care for |  |
| Impact of OFF periods on YOUR life |  |
| Management of OFF periods |  |

1. When you are in the doctor’s office, how often do you actually use the phrase “OFF period(s)” when talking about the symptoms that the person you care for experiences?
2. Never
3. Rarely
4. Sometimes
5. Often
6. Always
7. Does the doctor ask about the timing of medication (e.g., levodopa/carbidopa) at every, or almost every, visit?
   1. **IF YES:** Why do you think your doctor is interested in medication timing?

___FREE TEXT RESPONSE___

1. Using a scale from 1 to 5 (where 1=Strongly Disagree and 5=Strongly Agree) please rate your level of agreement with the following statements:
   1. I have difficulty discussing OFF periods with the doctor because I do not feel that he/she listens to what I have to say
   2. I have difficulty discussing OFF periods with the doctor because he/she is distracted by the computer/other technology during our clinic visits
   3. I have difficulty discussing OFF periods with the doctor due to lack of time at visits
   4. I have difficulty discussing OFF periods with the doctor because we do not use the same language to describe the issues.
   5. I have difficulty discussing OFF periods with the doctor because they are difficult to describe
   6. I am reluctant to tell the doctor how his or her OFF periods are really impacting me/us because I do not want to admit how much they are affecting me/us
   7. I am reluctant to tell the doctor about his or her OFF periods because I see it as a sign of progression of Parkinson’s disease.
   8. I am reluctant to tell the doctor how his or her OFF periods are really impacting me/us because I do not want my doctor to think I am complaining
   9. I am reluctant to tell the doctor about the OFF periods because they are variable/not consistent.
2. Do you feel that the doctor understands the burden, or impact, of OFF periods on the daily activities you have listed above?
   1. Yes
   2. No
   3. I do not know
3. Using the table below please indicate what strategies would help you to discuss OFF periods with the doctor:

| **Strategies** | **I have used this and it is helpful** | **I think this would be helpful if I tried it** | **This would not be helpful** |
| --- | --- | --- | --- |
| Having longer clinic visits |  |  |  |
| Having a free-flowing conversation with the doctor |  |  |  |
| Writing down a problem list/agenda to discuss with the doctor prior to visits |  |  |  |
| Answering a questionnaire about OFF symptoms at the clinic visit |  |  |  |
| Keeping a diary of OFF periods and medication timing prior to clinic visits |  |  |  |
| Sharing a video of OFF periods with my doctor at the visit |  |  |  |
| Having a wearable device record OFF periods automatically and transmit this information to the doctor |  |  |  |
| Other, please specify: ___ |  |  |  |

1. Have you communicated with the doctor between scheduled visits about OFF periods?
   1. No, it has not been necessary
   2. No, I would have liked to but the doctor is not available
   3. No, I would have liked to but I avoid bothering the doctor
   4. Yes

*If yes, “Has this been important to you?” (Yes/Somewhat/No)*

- 1. Not applicable – I am not the person who would communicate with the doctor.

1. Have you found any communication tools about OFF periods that you have found helpful? (Please describe and specify source) ______________________________
