# Supplementary material for: Communication About OFF Periods in Parkinson's Disease: A Survey of Physicians, Patients, and Carepartners
Source: Front Neurol. 2019 Aug 19;10:892. doi: 10.3389/fneur.2019.00892 (PMC6709650; doi:10.3389/fneur.2019.00892)
Supplement: Supplementary file 3 [file Data_Sheet_3.docx]

Communication about OFF periods in Parkinson’s disease Survey – Neurologists

**INTRODUCTION**

We are conducting a study about OFF periods in Parkinson’s disease. OFF periods (temporary

periods of poor mobility and increases in other symptoms) are common in Parkinson’s disease and associated with poorer quality of life. We aim to identify and better understand barriers and facilitators to communication about OFF periods between physicians, patients with Parkinson’s disease, and care partners. This quantitative survey was developed from the qualitative interviews that were conducted with 20 neurologists, seeking to understand to what extent the ideas expressed apply to the wider community of general neurologists and movement disorder specialists.

**If you are a general neurologist or a movement disorders specialist** and are interested in participating in a 10-15 minute survey on this topic please proceed.

S1 Which of the following best describes your specialty?

1. General neurologist
2. Movement Disorder neurologist
3. Other subspecialist neurologist (terminate)

S5 Approximately how many patients with Parkinson’s disease do you see in a month?

0-10 **[TERMINATE]**

**[TERMINATE]**

11-20

>20

S2 How much time do you typically have to assess a new patient with Parkinson’s disease

**<30 minutes**

**30-59 minutes**

**60 minutes or more**

On average, what percentage of your time is spent with a new patient with Parkinson’s Disease

____%

S3 How much time do you typically have to assess a follow-up patient with Parkinson’s disease?

Up to 15 minutes

16-29 minutes

30 or more minutes

S4 Since completion of your medical training, how many years have you been in clinical practice?

<6 years

6-10 years

>10 years

**EXPLANATION OF “OFF” PERIODS TO PATIENTS**

**OFF periods are periods when a patient’s Parkinson’s disease symptoms are not controlled by their medications, and symptoms temporarily return.**

1. At what point in the disease do you usually bring up the topic of “OFF” periods with patients?
   (a) At diagnosis
   (b) When initiating dopaminergic therapy
   (c) When patients start experiencing “off” symptoms
   (d ) Other, please explain: _____

Q1.1: How do you refer to “OFF” periods when speaking with your patients? (Click all that apply)

1. “OFF periods”
2. “Wearing off”
3. “OFF episodes”
4. “Fluctuations”
5. Other specific term or phrase (please specify) _________________________________
6. No specific term or phrase.
7. When you are discussing symptoms with patients, how often do you use the phrase “OFF” periods?
8. Never
9. Rarely
10. Sometimes
11. Often
12. Always
13. Which of the following symptoms do you identify as possible “off” symptoms? Check all that apply. **[RANDOMIZE OPTIONS]**
14. Slowness
15. Changes in mobility
16. Tremor
17. Pain
18. Sweating
19. Anxiety
20. Symptoms of REM-behavior sleep disorder
21. Constipation
22. Bladder dysfunction
23. Symptoms of impulse control disorder
24. Impaired sense of smell
25. Dyskinesia
26. How frequently do you discuss the following aspects of “OFF” periods at clinical visits:

**[INSTRUCTION]** "Please use the below scale, with "0" meaning “never" and "5" meaning "always"

**[RANDOMIZE OPTIONS]**

| **Aspects of “OFF” periods** | **[Likert scale] (1=never, 5=always)** |
| --- | --- |
| Motor symptoms (e.g., tremor, stiffness, slowness of movement) |  |
| Emotional symptoms (e.g., anxiety) |  |
| Symptoms of bodily function (e.g., urinary symptoms, sweating, hot flashes) |  |
| Impact of “OFF” periods on the patient’s life |  |
| Impact of “OFF” periods on the carepartner’s life |  |

1. In your current practice, if a non-motor symptom (e.g., pain, sweating, or anxiety) appears to fluctuate with timing of dopaminergic medication, would you typically adjust timing of medication to target this particular symptom?
   1. Yes
   2. No
2. In your current practice, please rate how frequently you use each method for assessing “OFF” periods in your patients who experience them.

**[INSTRUCTION]** "Please use the below scale, with "1" meaning “never" and "5" meaning "always"

**[RANDOMIZE OPTIONS]**

| **Method** | **Likert scale (1=never, 5=always)** |
| --- | --- |
| By speaking to patient during clinical interview |  |
| By speaking to care partner during clinical interview |  |
| Using a questionnaire that the patient and/or care partner completes prior to clinical interview |  |
| Using a motor diary that patient and/or care partner completes at home, prior to clinic visit |  |
| Levodopa challenge (observing a patient through a dose-response cycle of levodopa administration) |  |
| Using wearable technology (e.g., a device that collects data on patterns on movement of the patient at home, which may be analyzed for periods of tremor, lack of movement, or dyskinesia) |  |
| Other: _______ |  |

**BARRIER TO COMMUNICATING ABOUT “OFF” PERIODS**

1. Please rate the degree to which each of the following issues is a barrier to communication about “OFF” periods in your current practice.

**[INSTRUCTION]** "Please use the below scale, with "1" meaning “never" and "5" meaning "always"

**[RANDOMIZE OPTIONS]**

| **Barrier to communication regarding “off” periods** | **Likert scale** (1=not a barriers, 5=a major barrier) |
| --- | --- |
| Lack of time in clinical visits |  |
| Cognitive impairment in the patient |  |
| Cognitive impairment in the care partner (impacting the availability of collateral history) |  |
| Patient has difficulty recognizing his/her motor “OFF” symptoms |  |
| Patient has difficulty recognizing his/her non-motor “OFF” symptoms |  |
| Lack of a standard terminology/shared vocabulary for describing “OFF” periods |  |
| Confusion between tremor and dyskinesia in patient reporting of symptoms |  |
| Patient has poor understanding of the relationship between “OFF” periods and medication timing |  |
| Conflicting reports between patients and care partners of “OFF” symptoms |  |
| Patient reluctance to express the extent of his/her symptoms |  |
| Computer or technology used in documentation that distracts from the flow of the clinical interview |  |
| Treatment goals/expectations that are different between patients and yourself |  |
| My own knowledge related to “OFF” periods in Parkinson disease |  |
| Other: ______ |  |

**FACILITATORS TO COMMUNICATION ABOUT “OFF” PERIODS**

1. Rate the following according to their potential for facilitating the communication between you and your patient about “off” periods (5-point likert scale 1=would not be helpful, 5=very helpful)

**[RESPONDENTS BE ALLOWED TO SELECT “YES/NO” OPTIONS ONLY IF SCALE IS >=3]**

**[INSTRUCTION]** "Please use the below scale, with "1" meaning “never" and "5" meaning "always"

**[RANDOMIZE OPTIONS]**

| **Facilitator to communication regarding “off” periods** | **Likert scale (**5-point likert scale 1=would not be/is not helpful, 5=very helpful ) | **I use/have used this strategy (yes/no) (only for if 4 or 5 selected on Likert scale)** |
| --- | --- | --- |
| Presence of a care partner at the clinical visit |  |  |
| Free-flowing dialogue between yourself and patient and/or care partner during clinic visits |  |  |
| Access to multidisciplinary approach, with allied health personnel (e.g., nurse educators) |  |  |
| Information pamphlet explaining “OFF” periods to hand to patients and care partners |  |  |
| Diagram-based explanation of “OFF” periods to facilitate discussion with patients and care partners during clinic visits |  |  |
| On-line video explaining “OFF” periods for patients and care partners |  |  |
| Pre-consultation questionnaire to be completed prior to clinical interview |  |  |
| Paper-based motor diaries to be completed at home, prior to clinic visit |  |  |
| Digital app on smart phone for patients or care partners to record “OFF” time at home prior to clinic visit |  |  |
| Wearable technology to automatically and passively detect OFF time at home prior to clinic visit |  |  |
| Levodopa challenges (observing a patient through a dose-response cycle of levodopa administration) |  |  |
| Home videos provided by patients and/or care partners to illustrate a problematic “OFF” period symptom |  |  |
| Incorporating a teach-back method in communication during clinical visits (i.e., having patients and/or care partners repeat back information to ensure comprehension) |  |  |
| Small group classes for similar patient groups and their care partners (e.g., newly diagnosed patients), conducted by a nurse practitioner (or other allied health staff) |  |  |
| Repeated educational points over time (and multiple visits) |  |  |
| Other: ______ |  |  |
