# Supplementary material for: Communication About OFF Periods in Parkinson's Disease: A Survey of Physicians, Patients, and Carepartners
Source: Front Neurol. 2019 Aug 19;10:892. doi: 10.3389/fneur.2019.00892 (PMC6709650; doi:10.3389/fneur.2019.00892)
Supplement: Supplementary file 4 [file Data_Sheet_4.docx]

**Impact and communication about OFF periods**

This survey seeks to understand how patients and carepartners understand and communicate about OFF periods, and how OFF periods impact on patients and carepartners. The survey should take about 25 minutes to complete.

**Do you have Parkinson’s disease or are you the primary care giver for a person with Parkinson’s disease?**

1. **Yes I have Parkinson’s disease (direct to PD questionnaire)**
2. **Yes, I am the primary care giver for a person with Parkinson’s disease (direct to the carepartner questionnaire)**
3. **No (terminate)**

When a person with Parkinson’s disease benefits from medication, over time they can begin to experience episodes where the medications don’t work or don’t work as well. In those episodes those symptoms that are typically improved by the medication temporarily worsen. These episodes are called **OFF periods.**

1. Do you experience OFF periods, as just defined?
   1. Yes
   2. No **(TERMINATE)**
   3. I don’t know **(TERMINATE)**
2. Are you familiar with the term “OFF” or “OFF periods” related to Parkinson’s disease, as defined above?
   1. Yes
   2. No

**PERSONAL EXPERIENCE OF OFF PERIODS**

1. How many years ago did you begin to experience OFF periods?

a) less than1 year

b) 1 to 5 years

c) 6 to 10 years

d) More than10 years

e) I don’t know

1. Over the last week, on average, how many OFF episodes do you experience in a typical waking day?
   - Single Select (Radio)
     - No episodes, zero
     - 1 episode per day
     - 2 episodes per day
     - 3 episodes per day
     - 4 episodes per day
     - Greater than 4 episodes per day
     - I don’t know
2. Over the last week, on average, what is the typical duration of each OFF episode?
   - Single Select (Radio)
     - Less than 15 minutes
     - Between 15 and 30 minutes
     - Between 30 minutes and 45 minutes
     - Between 45 minutes and 1 hour
     - Between 1 hour and 2 hours
     - Greater than 2 hours
     - I don’t know

1. What proportion of your OFF periods come at unpredictable (i.e. unexpected) times?
   1. 0
   2. Less than 25%
   3. 25-50%
   4. More than 50%
   5. I don’t know
2. If >0: If the timing of your OFF periods were more predictable, how much would that lessen their impact on your life?
   1. Very much
   2. Somewhat
   3. Neutral
   4. Not at all
3. Do you currently keep track of your OFF periods using a paper or electronic record?
4. Yes
5. No

If yes, how do you keep track of your OFF periods (select all that apply)

1. Paper record
2. Electronic diary
3. Wearable device
4. Other method (please specify) ___________________________

**PERSONAL IMPACT OF OFF PERIODS**

1. Please check all of the symptoms that you experience during OFF periods in the table below,

| **Symptom** | **Yes/No/Unsure** |
| --- | --- |
| 1. Fatigue |  |
| 1. Sleepiness |  |
| 1. Tremor |  |
| 1. Stiffness |  |
| 1. Slowness of movement |  |
| 1. Change in gait/walking |  |
| 1. Increased falls |  |
| 1. Difficulty with hand coordination |  |
| 1. Difficult swallowing |  |
| i. Difficulty speaking |  |
| 1. Trouble breathing |  |
| 1. Nausea |  |
| 1. Pain |  |
| 1. Anxiety |  |
| 1. Irritability |  |
| 1. Agitation or restlessness |  |
| 1. Loss of motivation |  |
| 1. Sadness/depression |  |
| 1. Social withdrawal |  |
| 1. Hot flashes |  |
| 1. Sweating |  |
| 1. Loss of appetite |  |
| 1. Change in bladder function (e.g., urgency, incontinence) |  |
| 1. Difficulty thinking |  |
| 1. Other, please specify: ______ |  |

For each symptom indicated as “yes” need to follow with

A 5-point likert scale of impact:

1: No impact

5: Severe impact

1. In general, how much impact do the OFF periods have on your daily life?

5-point likert scale: 1=no impact, 5=severe impact

1. How much are each of the following aspects of YOUR life impacted by OFF periods? ( Need a likert scale for each symptom ranging from

1=no impact

To 5=severe impact)

|  |
| --- |
| Physical activity |
| Leisure/hobbies |
| Employment |
| Close Relationships |
| Friendships |
| Household tasks |
| Driving |
| Self-care/grooming |
| Independence |
| Communication |
| Your freedom to leave the home |
| Scheduled activities |

1. Using a scale from 1 to 7 (where 1=Strongly Disagree and 7=Strongly Agree) please rate your level of agreement with the following statements:
2. OFF periods frustrate me
3. OFF periods make me anxious
4. Having OFF periods is scary
5. Having OFF periods has hurt my self esteem
6. OFF periods make me feel embarrassed

**EDUCATION ABOUT OFF PERIODS**

1. How did you learn about OFF periods (select all that apply)
2. My doctor had previously told me about them
3. My doctor explained that they were OFF periods after I brought up the symptoms
4. My care partner/family explained it to me
5. I read about them on-line
   1. Which online source? ______
6. I read a book about Parkinson’s disease that explained what these symptoms were
   1. Which book? _____
7. My PD support group told me what was happening
8. I learned about it from a friend
9. Other, please specify: _____

1. Do you feel that you have had adequate education about OFF periods? (e.g. teaching from your doctor, or educational material made available to you)
   1. Yes
   2. No
2. What would be your preferred format for learning about OFF periods
   1. Explanation from the doctor
   2. Written handout or pamphlet
   3. Explanation from the doctor + written handout
   4. On-line video tutorial
   5. On-line written material material
   6. Other (please specify) ___________________________________-

**COMMUNICATION REGARDING OFF PERIODS**

The following questions relate to communication about OFF periods with the doctor who takes care of most of the Parkinson’s disease-related issues.

1. **What type of doctor primarily cares for your Parkinson’s disease?**

Movement Disorder specialist

General neurologist

Primary care physician

Geriatrician

Other

I don’t know

1. Do you discuss your OFF periods with this doctor at most (more than 50%) of the visits
   1. Yes
   2. No

**IF YES:** What aspects of OFF periods are discussed at most visits:

| **Aspects of OFF periods** | **Discussed with the doctor at most visits (Yes/no)** |
| --- | --- |
| Frequency |  |
| Motor symptoms (e.g., tremor, stiffness, slowness of movement) |  |
| Emotional symptoms (e.g., anxiety, depression) |  |
| Symptoms of bodily function (e.g., urinary symptoms, sweating, hot flashes) |  |
| Timing of OFF periods |  |
| Impact of OFF periods on your life |  |
| Management of OFF periods |  |

1. When you are in the doctor’s office, how often do you actually use the phrase ‘OFF period(s)’ when talking about the symptoms that you experience?
2. Never
3. Rarely
4. Sometimes
5. Often
6. Always
7. Does your doctor ask about what times of the day you take your Parkinson’s medication?
   1. **IF YES:** Why do you think your doctor is interested in medication timing?

___FREE TEXT RESPONSE___

1. Using a scale from 1 to 7 (where 1=Strongly Disagree and 7=Strongly Agree) please rate your level of agreement with the following statements:
   1. I have difficulty discussing OFF periods with my doctor because I do not feel that he/she listens to what I have to say
   2. I have difficulty discussing OFF periods with my doctor because he/she is distracted by the computer/other technology during our clinic visits
   3. I have difficulty discussing OFF periods with my doctor due to lack of time at visits
   4. I have difficulty discussing OFF periods with my doctor because we do not use the same language to describe the issues.
   5. I have difficulty discussing OFF periods with the doctor because they are difficult to describe
   6. I am reluctant to tell my doctor how my OFF periods are really impacting me/us because I do not want to admit how much there are affecting me/us
   7. I am reluctant to tell my doctor how my OFF periods are really impacting me/us because I do not want my doctor to think I am complaining
   8. I am reluctant to tell my doctor about my OFF periods because I see it as a sign of progression of Parkinson’s disease.
   9. I am reluctant to tell the doctor about my OFF periods because they are variable/not consistent
   10. I am embarrassed to admit that I experience OFF periods
   11. I don’t think to discuss OFF periods because I consider it as part of the life of a PD patient
   12. I forget to discuss OFF periods at my doctors’ visits
2. Do you feel that your doctor understands the burden, or impact, of OFF periods on the daily activities you have listed above?
   1. Yes
   2. No
   3. I do not know
3. Using the table below please indicate what strategies would help you to discuss OFF periods with your doctors:

| **Strategies** | **I have used this and it is helpful** | **I think this would be helpful if I tried it** | **This would not be helpful** |
| --- | --- | --- | --- |
| Having longer doctor visits |  |  |  |
| Having a free-flowing conversation with the doctor |  |  |  |
| Writing down a problem list/agenda to discuss with the doctor prior to visits |  |  |  |
| Answering a questionnaire about OFF symptoms at my doctor visits |  |  |  |
| Keeping a diary of my OFF periods and medication timing prior to clinic visits |  |  |  |
| Having a wearable device record OFF periods automatically and transmit this information to my doctor |  |  |  |
| Sharing a video of my OFF periods with my doctor at the visit |  |  |  |
| Having a partner at the visit to help describe the OFF periods |  |  |  |
| Other, please specify: ___ |  |  |  |

1. Have you communicated with your doctor between scheduled visits about your OFF periods?
   1. No, it has not been necessary
   2. No, I would have liked to but the doctor is not available.
   3. No, I would have liked to but I avoid bothering my doctor
   4. Yes

*If yes, “Has this been important to you?” (Yes/Somewhat/No)*

1. Have you found any communication tools about OFF periods that you have found helpful? (Please describe and specify source) ______________________________
